# 

**Published:** 1892-08-06

**Authors:** 


### CONTENTS.

The British Medical Association - - - - - 305

Passing Topics.—The Latest Criticism - - - - - 306

Medical History from the Earliest Times. By E. T. Withington, M.A., M.B. Oxon. XX.—Galen - - - 307

Everybody's Page - - - - - - - 308

The Meaning of Pain - - - - - - - 309

The Attitude of Great Writers towards Disease. VIII.—Dickens - - - - - - - - 310

Annotations.—Board School Teachers—A Popular Educator—A New Life Saving Apparatus - - - - - - 311

Notes and News - - - - - - - - 312

Around the Hospitals—Scraps and Gleanings - - 313

New Drugs, Appliances, and Things Medical—Disinfectants—Carbolic Acid and its Preparations - - - - 316

The Practitioners' Mirror and Retrospect—

Medicine.—Erysipelas - - - - - - - - 314

Surgery.—Spasmodic Torticollis: Treatment by Resection of Cervical Nerves - - - - - - - 314

Therapeutics.—Coronilla varia - - - - - - 315

The Institutional Workshop—

Metropolitan Hospital Sunday Fund - - - 317

Management.—The Invalid Children's Aid Association - 318

The Royal Hospital for Incurables, Putney - - 319

Hospital Opinion.—Finance - - - - - 320

Editor's Letter-Box.—"Lifeboat Saturday" - - - 320

Books Received - - - - - - - - 320

The Student of Medicine.—Appointments—Vacancies—Pass Lists.

### EXTRA SUPPLEMENT.—THE NURSING MIRROR.

En Passant.—Gravesend Nurses—St. Olave's District Nursing Association—Crieff Sick Nurse Association—Weston-Super-Mare—The Q.V.J.I.N.—Nursing of Out-door Paupers—Short Items—Well Done Aberdeen! - - - - - cxxix

The Management of Consumptive Patients. I.—The Infectiousness of Phthisis - - - - - cxxx

Experiences of Lowestoft Hospital - - - - - cxxxi

Wardmaids - - - - - - - - cxxxi

Our American Sisters (*concluded*) - - - - - cxxxii

Appointments - - - - - - - - cxxxiii

For Reading to the Sick.—Our Treasures - - - - cxxxiii

Everybody's Opinions.—Manners in the Midlands—Hospital and Asylum Nurses—Nursing at Carlisle Infirmary—Longton Cottage Hospital - - - - - cxxxiv

Lady Sanitarists - - - - - - - - cxxxv

Wants and Workers - - - - - - - cxxxv

Where to Go—Notes and Queries - - - - - cxxxv

The Real Mrs. Skeffington - - - - - - cxxxv

